# Supplementary material for: Foraging efficiency, social status and body condition in group-living horses and ponies
Source: PeerJ. 2020 Nov 9;8:e10305. doi: 10.7717/peerj.10305 (PMC7659649; doi:10.7717/peerj.10305)
Supplement: Supplemental Information 1 [file peerj-08-10305-s001.docx]

**Table S1** – breakdown of foraging time and observation time for each individual within the study herds

| **Herd Number** | **Herd Size** | **Mean percentage of time foraging** | **Mean observational time per individual (minutes)** |
| --- | --- | --- | --- |
| 1 | 3 | 80.67 | 68 |
| 2 | 2 | 64.00 | 79 |
| 3 | 4 | 92.50 | 56 |
| 4 | 3 | 58.67 | 49 |
| 5 | 6 | 75.17 | 32 |
| 6 | 7 | 51.86 | 26 |
| 7 | 10 | 64.90 | 25 |
| 8 | 9 | 92.00 | 20 |
| 9 | 8 | 93.88 | 35 |
| 10 | 7 | 68.00 | 34 |
| 11 | 9 | 60.89 | 27 |
| 12 | 4 | 100.00 | 38 |
| 13 | 6 | 63.00 | 35 |
| 14 | 10 | 95.00 | 32 |
| 15 | 3 | 73.00 | 70 |
| 16 | 5 | 96.40 | 49 |
| 17 | 7 | 76.71 | 37 |
| 18 | 5 | 62.20 | 40 |
| 19 | 5 | 67.20 | 40 |
| 20 | 3 | 90.33 | 79 |
