# Supplementary material for: Foraging efficiency, social status and body condition in group-living horses and ponies
Source: PeerJ. 2020 Nov 9;8:e10305. doi: 10.7717/peerj.10305 (PMC7659649; doi:10.7717/peerj.10305)
Supplement: Supplemental Information 2 [file peerj-08-10305-s002.docx]

**Table S2**: Statistically non-significant univariable associations (*p*>0.05) using mixed effects linear regression controlling for herd group and herd size as random effects

|  | **Unrewarded Active Behaviour Variable** | ***β*** | **S.E.** | **95% CI** | ***Z*** | ***p*** |
| --- | --- | --- | --- | --- | --- | --- |
| **Dominance Rank and Body Condition Score** |  | 0.66 | 0.29 | 0.09 – 1.24 | 2.27 | 0.03 |
|  |  |  |  |  |  |  |
| **Body condition and Unrewarded Active Behaviour** |  |  |  |  |  |  |
| **Frequency** | **Instances of moving whilst foraging** | -0.11 | 1.10 | -2.27 – 2.05 | -0.10 | 0.93 |
|  | **Instances of socially-mediated interference** | -0.78 | 1.53 | -3.77 – 2.22 | -0.51 | 0.61 |
|  | **Instances of displacement towards others** | 1.94 | 1.14 | -0.30 – 4.19 | 1.70 | 0.09 |
|  | **Instances of scratching** | -5.67 | 3.40 | -12.34 – 0.99 | -1.67 | 0.10 |
|  | **Instances of startle** | -11.50 | 19.62 | -49.95 – 26.95 | -0.59 | 0.55 |
|  |  |  |  |  |  |  |
| **Dominance Rank and Unrewarded Active Behaviour** |  |  |  |  |  |  |
| **Frequency** | **Instances of vigilance** | -0.06 | 0.09 | -0.23 – 0.11 | -0.65 | 0.51 |
|  | **Instances of scratching** | -0.67 | 1.03 | -2.69 – 1.35 | -0.65 | 0.51 |
|  | **Instances of startle** | -1.09 | 5.93 | -12.70 – 10.52 | -0.18 | 0.88 |
